# New CD20 alternative splice variants: molecular identification and differential expression within hematological B cell malignancies

**DOI:** 10.1186/s40164-016-0036-3

**Published:** 2016-03-01

**Authors:** Clémentine Gamonet, Elodie Bole-Richard, Aurélia Delherme, François Aubin, Eric Toussirot, Francine Garnache-Ottou, Yann Godet, Loïc Ysebaert, Olivier Tournilhac, Dartigeas Caroline, Fabrice Larosa, Eric Deconinck, Philippe Saas, Christophe Borg, Marina Deschamps, Christophe Ferrand

**Affiliations:** 1 INSERM UMR1098, Établissement Français du Sang Bourgogne Franche Comté, Université de Franche-Comté, SFR FED4234, 25020 Besançon, France; 2 EA3181 et Service de Dermatologie, Université de Franche Comté, CHU de Besançon, Besançon, France; 3 CHRU, Department of Rheumatology, Université de Franche-Comté EA 4266, INSERM CIC-1431, 25000 Besançon, France; 4 EA 4266, Université de Franche-Comté, Besançon, France; 5 Inserm U1037, Université Toulouse 3-ERL CNRS, CHU Purpan, Toulouse, France; 6 Hématologie Clinique, CHU Estaing, 1 Place Lucie Aubrac, 63003 Clermont-Ferrand Cedex 1, France; 7 Hematologie, CHU Bretonneau, Tours, France; 8 Hematology Department, CHU Jean Minjoz, 25020 Besançon, France; 9 Laboratoire de Thérapeutique Immuno-Moléculaire et cellulaire des cancers, INSERM UMR1098, Etablissement Français du Sang–Bourgogne/Franche-Comté, 8, rue du Docteur Jean-François-Xavier Girod, 25020 Besançon Cedex, France

**Keywords:** CD20, Alternative splicing, B malignancies, EBV transformation, CLL

## Abstract

**Background:**

CD20 is a B cell lineage–specific marker expressed by normal and leukemic B cells and targeted by several antibody immunotherapies. We have previously shown that the protein from a CD20 mRNA splice variant (D393-CD20) is expressed at various levels in leukemic B cells or lymphoma B cells but not in resting, sorted B cells from the peripheral blood of healthy donors.

**Results:**

Western blot (WB) analysis of B malignancy primary samples showed additional CD20 signals. Deep molecular PCR analysis revealed four new sequences corresponding to in-frame CD20 splice variants (D657-CD20, D618-CD20, D480-CD20, and D177-CD20) matching the length of WB signals. We demonstrated that the cell spliceosome machinery can process ex vivo D480-, D657-, and D618-CD20 transcript variants by involving canonical sites associated with cryptic splice sites. Results of specific and quantitative RT-PCR assays showed that these CD20 splice variants are differentially expressed in B malignancies. Moreover, Epstein–Barr virus (EBV) transformation modified the CD20 splicing profile and mainly increased the D393-CD20 variant transcripts. Finally, investigation of three cohorts of chronic lymphocytic leukemia (CLL) patients showed that the total CD20 splice variant expression was higher in a stage B and C sample collection compared to routinely collected CLL samples or relapsed refractory stage A, B, or C CLL.

**Conclusion:**

The involvement of these newly discovered alternative CD20 transcript variants in EBV transformation makes them interesting molecular indicators, as does their association with oncogenesis rather than non-oncogenic B cell diseases, differential expression in B cell malignancies, and correlation with CLL stage and some predictive CLL markers. This potential should be investigated in further studies.

**Electronic supplementary material:**

The online version of this article (doi:10.1186/s40164-016-0036-3) contains supplementary material, which is available to authorized users.

## Background

CD20 protein was highlighted in 1980 as a B lymphocyte–specific cell-surface antigen expressed in all stages of B cell ontogenesis except for early pro-B cells and plasma cells [1]. Despite no identified ligands, CD20 functions were investigated, and studies assigned it a role in cell differentiation [2] and calcium flux pathways [3].

The anchorage within the membrane of the 33 kDa protein makes it a good candidate as an ion channel [3], especially when organized into tetramers [4]. Moreover, the presence of two extracellular loops allows for targeting by monoclonal antibodies (MAbs) to induce B cell depletion. The most well-known MAb is rituximab (RTX), which has greatly improved treatment of B cell malignancies [5], in association or not with chemotherapy [6]. After RTX, numerous other MAbs (such as obinutuzumab and ofatumumab) were subsequently developed to improve B cell depletion but also to treat RTX resistance to or escape from treatment [7].

CD20 is encoded by a MS4A family gene located on chromosome 11. Multiple transcription initiation sites have been identified, and the translated region of this gene is located between the third (193th nucleotide) and eighth exons (216th nucleotide), resulting in a coding sequence of 894 bp distributed into six exons [8]. Moreover, alternative splicing of the CD20 gene has been highlighted, occurring in the 5′ untranslated region and resulting in translation of three alternative CD20 mRNAs encoding the same protein in human B lymphocytes.

Alternative splicing remains a key process of pre-RNA maturation and allows an increase in protein translation and phenotype diversity [9]. Different patterns of splicing have been described, based on two families of regulatory proteins (constituting the spliceosome), the serine-rich (SR) and heterogeneous nuclear ribonucleoproteins (hnRNP) (for review, see [10]).

Aberrant splicing, caused by mutation in splice site sequences within cancer-related genes or in genes encoding splicing regulation proteins [11], has a dominant role in tumor establishment, progression, and response to treatment [12]. Abnormal splicing mechanisms produce numerous cancer-associated alternatively spliced variants that could promote angiogenesis, invasion, and drug resistance, conferring a more aggressive tumoral profile [13]. These alternative variants are differentially expressed in tumors [14] and thus may be used as diagnostic tools and prognostic markers [15]. Moreover, emerging treatments target new isoform proteins encoded from aberrant splicing [16] or modify splice site selection by oligonucleotide approaches to prevent abnormal splicing [17].

In oncohematology, numerous spliceosome gene mutations have been identified in chronic lymphocytic leukemia (CLL), myelodysplastic syndromes, and lymphomas; among the most well-known of these are those involving SF3B1, U2AF1, and SRSF2 [18–20]. Alternative splicing occurring in B cells could also be modified by Epstein–Barr virus (EBV) infection in which the BMLF1 viral protein modifies STAT1 splicing after binding with the spliceosome component SRp20 [21] and thus may influence immortalization of target B cells.

We [22] and others [23] have identified novel alternative CD20 transcripts, fully matching the MS4A1 sequence, except for 501 bp (from nucleotides 111–612, starting +1 of the ATG codon) flanked by the cryptic acceptor (AS) and donor (DS) splice sites. The resulting in-frame cDNA sequence encodes a truncated CD20 protein, called D393-CD20 (previously named ΔCD20 [22]), that is missing the major part of the transmembrane and extracellular domains, including the RTX epitope. Interestingly, this protein has been observed in malignant or EBV-transformed B cells but not in peripheral blood mononuclear cells (PBMCs), bone marrow–derived mast cells, or plasmocytes from healthy donors.

Additional investigations of D393-CD20 protein expression by western blotting on different hematologic samples have allowed us to detect extra signals that we followed up in the current work, extended to autoimmune or EBV-infected samples. Our molecular analysis has led to the description and characterization of new alternative CD20 transcripts that are differentially expressed in hematologic malignancies.

## Results

### Additional band signal is detected by c-terminal CD20 western blotting on blood samples collected from patients with hematologic malignancies

**Fig. 1:**
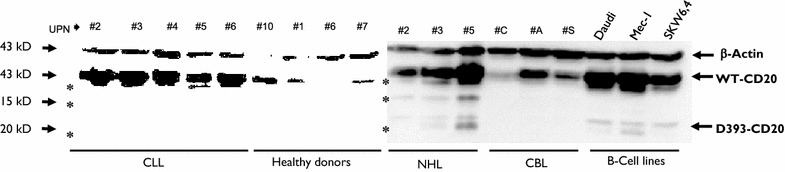
Western blot of CD20 expression. Western blotting of different samples collected from patients carrying B malignancies. *CLL* chronic lymphoid leukemia, *NHL* non-Hodgkin lymphoma, *CBL* cutaneous b lymphoma. Three B cell lines were included as well as samples from healthy donors as controls. β-actin was used as a protein-loading control. CD20 expression detection was performed using a C-terminal polyclonal CD20 antibody. (*) showed additional bands excluding wt- and D393-CD20 protein signals. Chemiluminescence time exposure was 5 min

As expected, western blot analysis using a carboxy terminus CD20 antibody targeted to circulating PBMCs from patients with B cell hematologic malignancies (CLL and NHL), CBL, B cell lines, or healthy donors revealed immunoreactive bands at 35 kDa corresponding to the full-length CD20 protein, indicating the presence of B lymphocytes in each sample (Fig. 1). As previously described, a band at 19 kDa, encoded by the CD20 alternative transcript D393-CD20 [22], was detected on CLL (5/5) and NHL samples (3/3), as well as on leukemic B cell lines (3/3). In contrast, the three CBL (without tumoral circulating B cells, as detected by B cell clonality analysis) and the four healthy donor samples were all negative for the 19 kDa band.

We clearly detected an unexpected additional immunoreactive band at approximately 27 kDa in all CLL and NHL samples. This band was also detected on MCL samples (data not shown). Surprisingly, this band was not detected on the three B cell lines. Moreover, western blot allowed detection of a supplementary signal at 33 and 17 kDa, respectively, close to the 35 kDa (full-length CD20 protein) or the 19 kDa (D393-CD20) bands.

### Both CD20 homologous and truncated nucleotide sequences are identified in B cell lines

After RT-PCR of the full-length CD20 (fl-CD20) coding sequence, agarose gel electrophoresis allowed us to detect the expected two 894 and 393 bp PCR products corresponding respectively to the wt- and D393-CD20 cDNA sequences. None of these visible amplified DNA fragments matched in size to products that could correspond to a sequence encoding the 27 kDa or other additional signals.

All fl-CD20 PCR fragments between <894 and 100 bp in length, excluding the major 393 bp PCR product, were gel purified, TA cloned, amplified, and Sanger sequenced. Sequencing of more than 150 individual bacterial colonies allowed identification, in addition to the D393-CD20 sequence, of four new nucleotide sequences partially homologous to the wtCD20 reference nucleotide sequence published in GenBank (NM152866.2) (Additional file 1: Figures S2 and S3). The four sequences are named according to the length of the nucleotide deletion compared to the CD20 reference. Thus, D657-CD20, D618-CD20, D480-CD20, and D177-CD20 indicate deletions of 237, 276, 414, and 717 bp, respectively.

### All newly identified sequences code for in-frame CD20 transcript variants resulting in MS4A1 alternative splicing

**Fig. 2:**
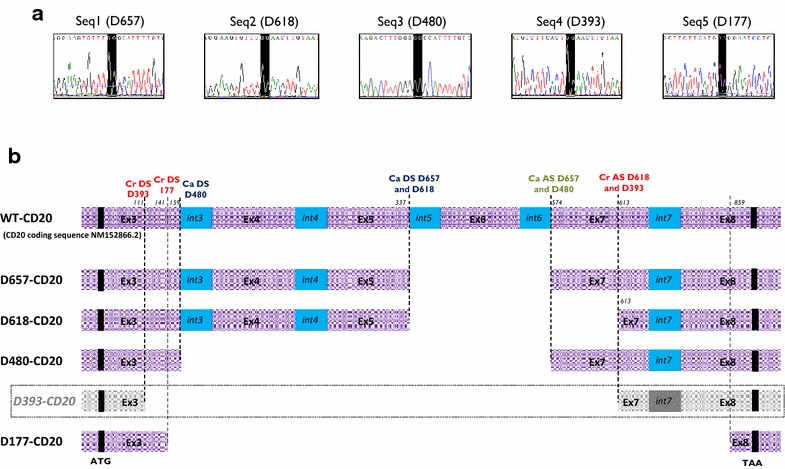
Characterization of new alternative CD20 transcript sequences. **a** Sequencing electropherograms showing junction areas resulting from alternative splicing number in brackets indicate size length deletion in nucleotides compared to the wtCD20 reference coding sequence. **b** Schematic alignment of newly identified sequences with wtCD20, reporting sequence deletions. Previously D393-CD20 sequence is framed. Canonical (Ca) or cryptic (Cr) donor (DS) or acceptor (AS) splice sites are reported as well as their nucleotide position from +1ATG nucleotide (in *italics*)

Bioedit© alignments revealed that all of the new sequences matched perfectly at the 5′ and 3′ regions with the conservation of start and stop codons of the wtCD20 whereas we detected a missing central area, generating a new sequence junction (Fig. 2a, b). A deeper analysis of the fusion sequences allowed highlighting of an alternative splicing phenomenon, bringing in a combination of cryptic or canonical DS or AS sites. Five splicing sites corresponded to canonical and three to cryptic sites, either DS or AS. Identification of the three cryptic-DS or cryptic-AS was confirm using the online splicing prediction tools *SplicePort Prediction* [24] and *ASSP Prediction* [25] (Fig. 2b).

Based on [26], two patterns of splicing involving both canonical DS and AS were identified as exon or multiple exon skipping for D480- and D657-CD20, respectively. One splicing pattern involving cryptic and canonical sites was qualified as an alternative 3′ splice site (D618-CD20); lastly, two patterns (including the known D393-CD20) concerned alternative 5′ and 3′ cryptic splice sites. The characteristics of the splice variant transcripts are reported in Fig. 2b and summarized in Table 1. All sequences were in frame, and translation generated new amino acid fusion sequences (Fig. 2c).

**Table 1:** Summary of characteristics of CD20 splice variant transcripts

Transcripts	Donor site (DS)				Acceptor site (AS)				Reading frame	CDS (length) (bp)	Putative proteine		Junction sequence	
	nt^a^	Cryptic (Cr) or canonical (Ca)	Location	Seq	nt^a^	Cryptic (Cr) or canonical (Ca)	Location	Seq			AA (length)	Size (kDa)	nt	AA
D393-CD20	112	Cr	Ex3	GT	612	Cr	Ex7	AG	Yes	393	131	17–23 (15)	TTCACTG/GAACTTG	RMSSL/ELVIA
D657-CD20	337	Ca	*Int*5	GC	573	Ca	*Int*6	AG	Yes	657	219	25	GTGTTTG/GGCATTT	SRKCL/GILSV
D618-CD20	337	Ca	*Int*5	GC	612	Cr	Ex7	AG	Yes	618	206	23	GTGTTTG/GAACTTG	SRKCL/ELVIA
D480-CD20	160	Ca	*Int*3	GT	573	Ca	*Int*6	AG	Yes	480	160	18	TTTGGGG/GGCATTT	SKTLG/GILSV
D177-CD20	142	Cr	Ex3	GA	858	Cr	Ex8	AG	Yes	177	59	6,5	CATGAGG/GAATCCT	SFFMR/ESSPI

^a^From ATG site: ref Genebank NM 152866.2

### Design of RT-PCR and RT-qPCR molecular tools allowed for specific detection and quantification of all newly identified spliced CD20 sequences

**Fig. 3:**
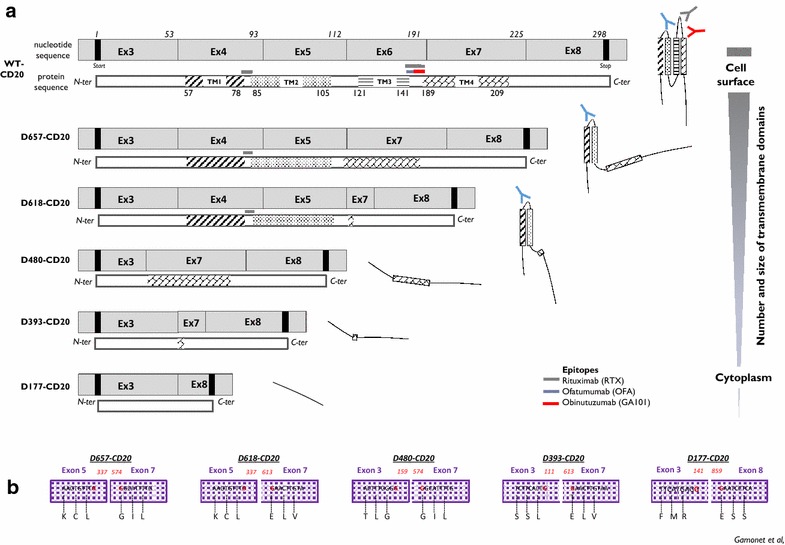
Alternative CD20-transcripts, putative proteins and junction characterization. **a** Schematic representation of CD20 variant coding transcripts. Transmembrane domains (TM) are positioned on the linear N-ter/C-ter protein, as well as position of the main clinical anti-CD20 antibody epitopes. Rituximab (*gray line*), Ofatumumab (*blue line*) and Obinutuzumab (*red line*). Schematic representation of antibody recognition on the putative CD20 variant-proteins. AA position is provided and numbered from the first ATG (Met) start codon. **b** Schematic reconstitution of amino acidic (AA) junction area after splicing involving canonical (*closed boxes*) or cryptic (*open boxes*) splice. AA sequence flanking junction is provided as well as AA position

To study the presence of transcripts and their level of expression, we designed RT-PCR and RT-qPCR assays (Additional file 1: Figure S1). As shown in Fig. 3a, fl-CD20 PCR allowed amplification of all CD20 alternative transcripts from either genomic DNA or cDNA extracted or synthesized from transfected PG13 cell lines. Moreover, transcript-specific RT-PCR allowed detection specifically of the respective CD20 alternative transcripts without cross-reactivity with the others, as shown when the target used was gDNA (Fig. 3b). Interestingly, D393spe-PCR amplified cDNA synthesized from ARN extracted from wt-, D657-, D480, and D393-CD20 cell lines. Positive signals detected with D177spe-PCR from all cell lines meant that all constructs could produce the D177-CD20 transcript.

Finally, RT-qPCR assays allowed specific detection without cross-reactivity (data not shown) from one CD20 transcript variant to another. Reproducibility of all assays was assessed in seven independent experiments on four different B cell lines: Raji, Mec, Rec, and SKW6.1 (Fig. 3c). Standard deviations for values from each RT-qPCR for all four cell lines were (min–max) (0.52–0.8), (0.05–0.2), (0.1–0.52), (0.001–0.39), and (0.28–0.62), respectively, for D657-, D618-, D480-, D393-, and D177-CD20 PCR. Interestingly, we noted that B cell lines resulting from different B cell malignancies present specific CD20 splicing profiles.

### Reintroduction of intron sequences within the coding CD20 sequence confirms involvement of canonical DS or AS splicing sites in D657-, D618, and D480-CD20 splice variant transcription

**Fig. 4:**
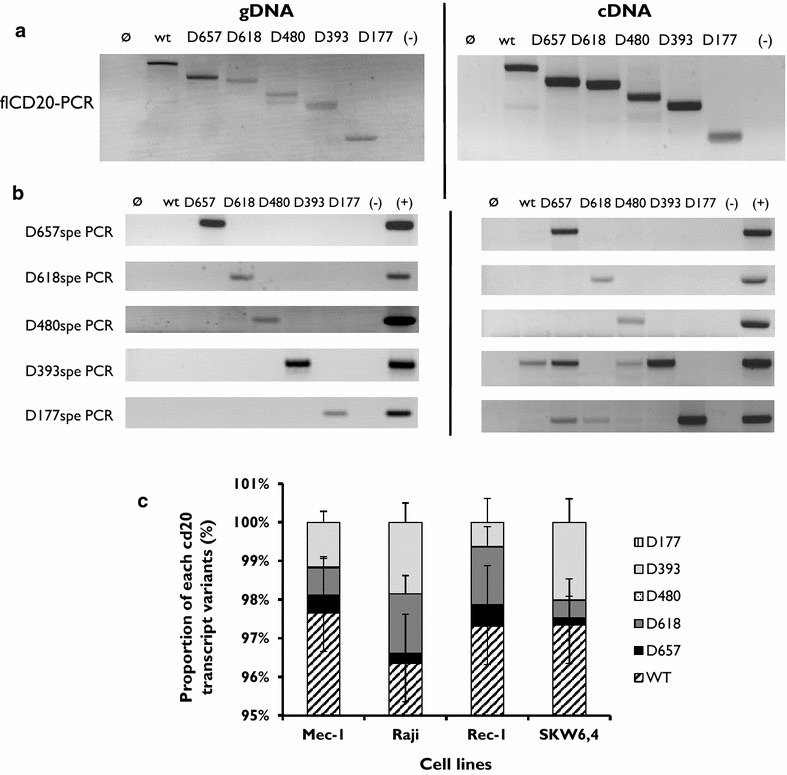
RT-PCR and RT-qPCR assays of different CD20 transcript variants. **a** Full-length PCR (fl-CD20) allowed amplification of all CD20 alternative transcripts. Genomic DNA (gDNA) from wild-type PG13 (ø) and PG13 transfected by wtCD20, D657-, D618-, D480-, D393-, or D177-CD20 were amplified using primers specific for the 5′ (start codon) and 3′ (stop) CD20 gene regions, common to the six transcripts. H_2_O was used as negative control (−), and the plasmid carrying the specific CD20 variant was added to the positive control (+). **b** CD20 variant-specific PCR was designed to amplify each alternative transcript specifically. **c** Proportion (in %) of each CD20 variant transcript in four different B cell lines. Means and SD calculated from seven independent experiments of RT-qPCR quantification

To confirm that canonical sites associated with cryptic splicing sites may be involved in CD20 variant transcription, as hypothesized from sequencing identification, some intron (3, 5, 6) sequences were used to generate artificial constructs carrying intron sequences within the wtCD20 coding sequence (Fig. 4a). D393- and D177-CD20 were produced by all three constructs independently of the presence of canonical sites because splicing involved only cryptic DS and AS. However, reintroduction of int5 alone in addition produced D618-CD20 transcripts. Dual reintroduction of int3 and 6 produced D480-CD20 whereas the presence of int5 and 6 allowed expression of D657- and D618-CD20 mRNA (Fig. 4b).

All of these results confirmed that the cell spliceosome machinery can process the ex vivo D480-, D657-, and D618-CD20 transcript variants by involving canonical sites associated with cryptic splice sites.

### EBV transformation modifies the CD20 splicing profile and increases mainly D393-CD20 variant transcripts

Among the four B cell lines, CD20 splicing quantification showed a higher and significant increase in D393-CD20 variants in the EBV-transformed cell line SKW6.4. For this reason, the impact of EBV infection or transformation on CD20 splicing was investigated within different kinds of EBV samples.

**Fig. 5:**
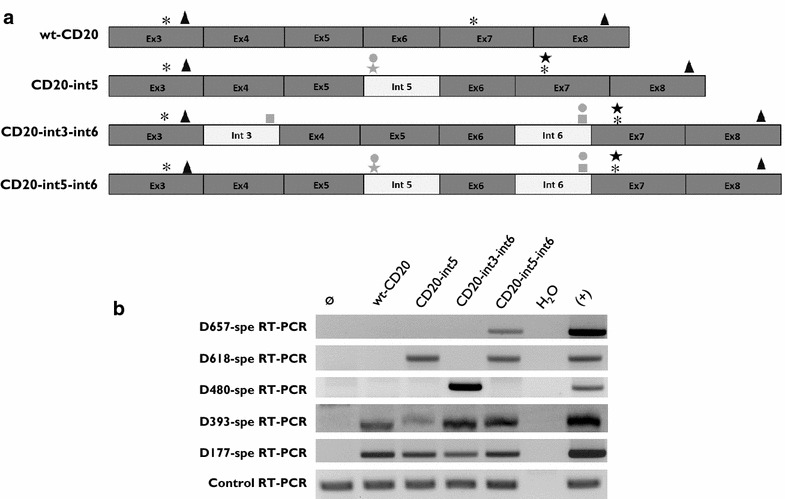
Intron reintroduction within the wtCD20 coding sequence. **a** Schematic representation of position of alternative splice sites (ASS) in the wtCD20 coding sequence and within the constructs after reintroduction of introns 5 (CD20-int5), 3 and 6 (CD20-int3–int6), and 5 and 6 (CD20-int5–int6). ASS positions for D657 (*closed circle*), D618 (*closed star*), D480 (*closed square*), D393 (*), and D177 (*closed triangle*) are indicated. *Gray* and *black symbols* represent canonic and cryptic splice sites, respectively. **b** Specific RT-PCR detection of different CD20 variants in transfected HT1080 cell lines with different constructs. Plasmid was used as positive control and untransfected HT1080 cells as negative (ϕ). Raf amplification PCR was used as control for the cDNA synthesis

Six EBV-transformed BLCLs were derived from the PBMCs of six healthy donors. As shown in Fig. 5a, total CD20 splicing was significantly increased in BLCL (3.4-fold, p < 0.01) compared to their respective PBMCs. Separate CD20 splice variant analysis revealed that increased total CD20 splices involved D657- and D618-CD20 but mainly and statistically significantly D393-CD20 (110 time, p < 0.001). D393-CD20 represented the major part (76.5 %) of the total CD20 splice increase compared to both D657- and D618-CD20 (23.4 %).

In contrast, total CD20 splicing did not vary significantly either for IMN samples compared to healthy PBMCs (Fig. 5b) or for EBV-reactivated samples after allograft (Fig. 5c), although we noted an increase in D657 and D618-CD20 splicing. Interestingly, D393-CD20 transcripts did not increase in these EBV-infected cases compared to EBV-transformed cell lines.

### CD20 splice variant profile expression can discriminate B cell malignancies

**Fig. 6:**
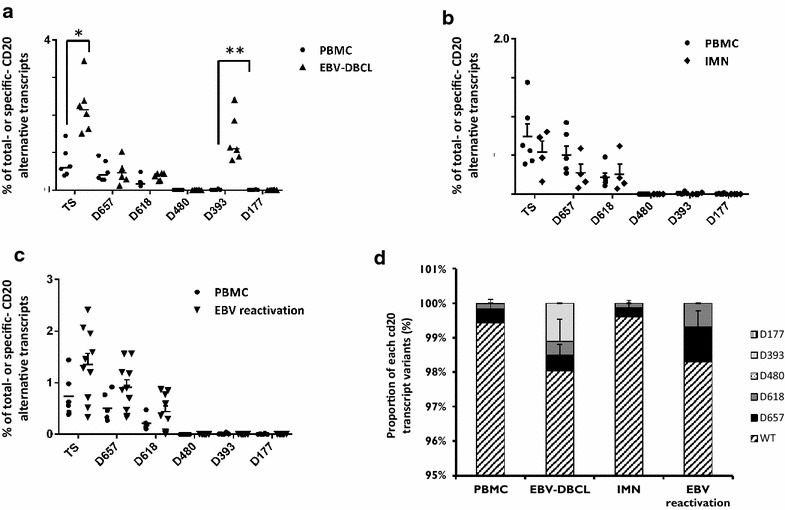
CD20 splicing quantification of EBV samples. Quantification by RT-qPCR of total (TS) and specific CD20 splice variants of **a** EBV-transformed B lymphoblastoid cell line, BLCL (*closed triangle*), compared to their respective PBMCs (*closed circle*); n = 6. **b** Infectious mononucleosis (IMN, *closed rhombus*, n = 4); **c** EBV-reactivated samples (EBV load increase >2 Log/ml. EBVr, inverted *closed triangle*, n = 10) after allograft compared to heathy PBMCs (*closed circle*, n = 6). (*) and (**) are the results of X^2^ tests with p < 0.02 and p < 0.002, respectively. CD20 transcript quantification within PBMCs reported as reference in all total or specific splicing quantification analyses. **d** Proportion (in %) of each CD20 transcipt variant in EBV samples in comparison with PBMC

Using the different CD20 variant profiles in the different B cell lines, Raji, Mec, Rec, and SKW6.1 (Fig. 3c), derived from different hematologic diseases (respectively, CLL, Burkitt lymphoma, MCL, B lymphoblastoid), we investigated the RT-qCPR expression of the different CD20 variant transcripts in different B cell malignancies. The percentage of total alternative CD20 transcripts from all four B cell lines but also from the primary cells of FLs and DLBCLs were significantly different from healthy PBMCs (p = 0.01 and <0.01 respectively) (Fig. 6a).

When analyzed separately, D657 was found to be mainly involved in the increase of total CD20 splicing (81.93 %) whereas D618 and D393 represented, respectively, 7.04 and 10.66 % of the increase in FL. In contrast, in DLBCLs, the increase was due in part to D657-CD20 (43.87 %) but also to D393-CD20 (40.26 %) whereas D618 participated only at 15.72 % in the increase (Fig. 6b).

### Relevance of alternative CD20 splice variant quantification within three different CLL patient cohorts

**Fig. 7:**
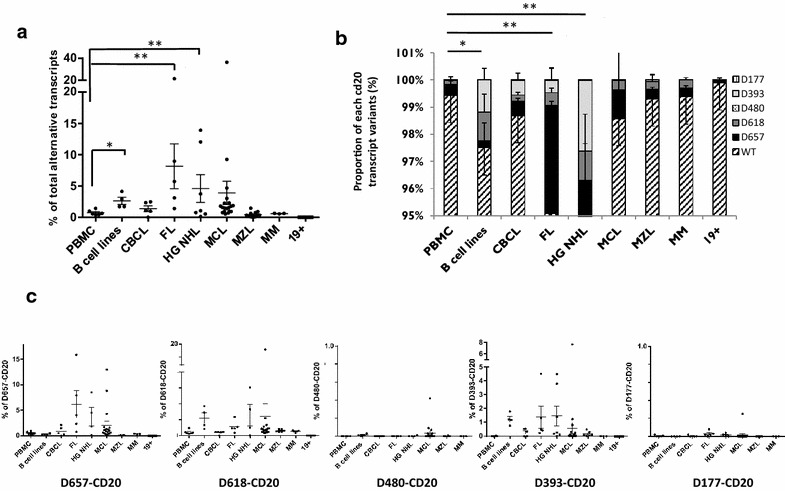
RT-qPCR quantification of all CD20 splice variants within B cell malignancies. Quantification by RT-qPCR of total **a** or specific **b**, **c** CD20 splice variants in four B cell lines, in different B cell malignancies, compared to PBMCs from healthy donors (n = 6). **c** Proportion (in %) of each CD20 transcript variant in different B cell malignancies. *CBCL* cutaneous B cell lymphomas (n = 5), *FL* follicular lymphoma (n = 5), *DLBCL* diffuse large B cell lymphoma (n = 5), *MCL* mantle-cell lymphoma (n = 19), *MZL* marginal zone lymphoma (n = 12), *MM* multiple myeloma (n = 3). (*) and (**) are the results of X^2^ tests with p = 0.01 and p < 0.001, respectively, compared to PBMC samples

**Fig. 8:**
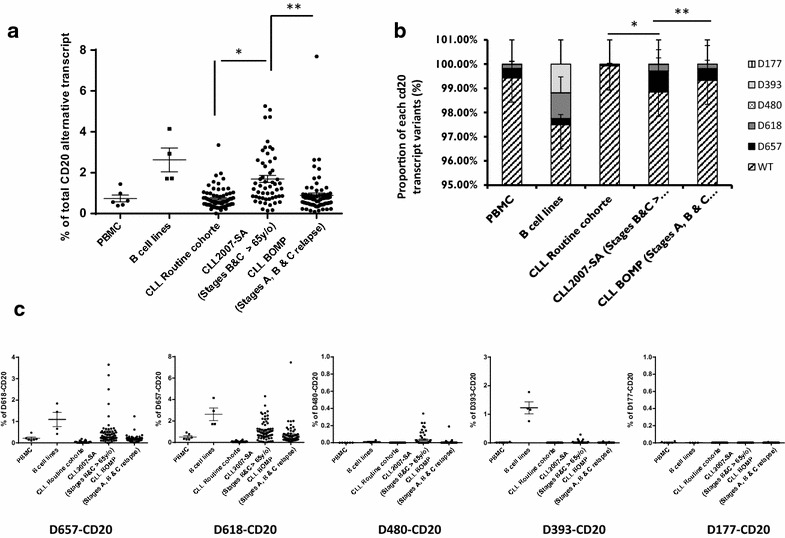
RT-qPCR quantification of total or specific CD20 splice variant expression within three different CLL sample cohorts. Quantification by RT-qPCR of total **a** or specific **b**, **c** CD20 splice variants in three cohorts of patients: CLL patient samples collected during routine diagnosis (50.8–49.1 %, stages A–B/C, respectively) (CHU Toulouse, France, n = 70); CD19-positive B cells purified from diagnostic stages B and C (65.5 and 34.5 %, respectively); and CLL samples from elderly patients (>65 years; median, 71.2) (CLL2007-SA trial (n = 54) or from patients with relapsed stages A, B, or C (mainly stages B and C for 88.7 %) with active disease (CLL01 BOMP clinical trial, n = 70). **c** Proportion (in %) of each CD20 transcript variant in different CLL cohorts. (*) and (**) are the results of X^2^ tests with p = 0.01 and p < 0.001, respectively, compared to PBMC samples

We took advantage of the availability of CLL samples from three different cohorts of patients (two collected at diagnosis and one at relapse): routinely collected CLL patient samples (50.8–49.1 % stages A–B/C respectively) for routine diagnostic analysis (CHU Toulouse, France, n = 70); CD19-positive B cells purified from diagnostic stage B and C (65.5 and 34.5 %, respectively) CLL samples from elderly patients (>65 years, median 71.2) (CLL2007-SA trial [27], n = 54); and samples from relapsed stage A, B, or C (mainly stages B and C, 88.7 %) active-disease patients (CLL01 BOMP clinical trial, n = 70). Characteristics of CLL populations are given in Additional file 1: Table S2. Considering the percentage in CLL2007-SA, the median of total CD20 splicing (1.26 ± 1.23 %) was significantly higher than in routine CLL (0.65 ± 0.5 %, p < 0.02) or BOMP (0.76 ± 1.02 %, p < 0.001) (Figs. 7a, 8). The increase was mainly due to the D657- and D618-CD20 transcript variants and to a lesser degree to the D393-CD20 transcripts (Fig. 7b).

## Discussion

We have previously identified a novel alternative CD20 transcript, fully matching the MS4A1 sequence, except for a 501-bp region flanked by cryptic AS and DS [1]. The resulting in-frame cDNA sequence encodes a truncated CD20 protein revealed by a C-terminal CD20 polyclonal antibody. Interestingly, this protein has been observed in malignant or EBV-transformed B cells but not in PBMCs, bone marrow–derived mast cells, or plasmocytes from healthy donors.

Detection of unexpected additional western blot signals with an anti-carboxy terminus CD20 antibody led us to conduct a deep molecular analysis to characterize potential transcripts that could match the protein signal. Starting from nucleic acid material extracted from B cell lines or, interestingly, from primary samples of patients with B cell hematologic diseases such as CLL, MCL, or DLBCL, we identified and characterized more precisely, in addition to the D393-CD20, four additional CD20 splice variants. Two of these variants are the result of exon skipping (D657- and D480-CD20), and the other two result from the use of alternative splice sites: a canonical DS and a cryptic AS for D618-CD20 and two cryptic splice sites for D177-CD20, by previously well-described mechanisms [10]. In addition to the D393-CD20 transcript, Small et al. [23] have already detected the D618- and D657-CD20 transcripts only in lymphoblastoid B cell lines. In the current work, we detected expression of these two transcripts in primary samples of human B cell diseases and reported for the first time two novel additional D480- and D177-CD20 transcripts. We also demonstrated ex vivo the involvement of canonical sites associated with cryptic splice sites that produce these transcripts. Although it should be formally demonstrated, except for D177, the lengths of the D393, D618-, D657-, and D480-CD20 transcripts matched the immunoreactive bands on western blot.

We designed quantitative molecular tools for studying alternative CD20 transcript expression in different B autoimmune, malignant B diseases or EBV-infected samples.

The comparison of splicing profiles revealed a more important CD20 alternative splicing in B diseases compared to healthy donors, suggesting a splicing deregulation in these pathologies.

Whereas a slight increase of CD20 alternative splicing was detected in CBCL, LZM, MM, and some CLL samples, a significantly higher amount of alternative transcripts was observed in FL, HG-NHL, and EBV-transformed B cell lines. In all cases, the increase compared to healthy donors results from a higher proportion of D618- and D657- CD20 transcripts. In addition, this increase is associated with a D393-CD20 expression induction in lymphomas (FL, DLBCL, Burkitt and MCL) and EBV transformation. Interestingly, in autoimmune diseases (rheumatoid arthritis and pemphigus), we never detected D393-CD20 transcripts [28, 29]. These observations suggest a splicing deregulation during oncogenesis leading to D393-CD20 expression, which could be an interesting molecular marker of B malignancies.

From another side, the slight increase of D657- and D618-CD20 expression could be the result of splicing deregulation associated with an enhanced proliferation and activation [30] during cancer but also during autoimmune disease and virus infection [31].

Increase of D393-CD20 occurs mainly in post-germinal center (GC) lymphomas (FL, Burkitt, and DLBCL). In GC, BCR maturation requires activation-induced cytidine deaminase (AID) intervention to introduce mismatches, which are then repaired by a mismatch repair complex. This process is known to be responsible for genetic abnormalities involved in oncogenesis but could also disturb splicing. Indeed, interactions and associations have been identified between AID and splicing factor SnRNPs such as U2AF65 [32], PTB2, and SRSF2 [33]. This link may explain in part how the AID activation pathway could lead to deregulation of splicing factors that disturb CD20 splicing, thus producing alternative CD20 variant expression in post-GC lymphomas. In post GC CLL, the fact that D393-CD20 increase was not observed may be explained by a lower AID expression [34] and activation.

Splicing pattern of immortalized B cell after EBV virus infection (DBCL) revealed a significantly greater increase in total CD20 splice variants, mainly because of an expression of D393-CD20. Interestingly, CD20 splicing was not statistically increased in IMN or in reactivated EBV samples: although we noted an increase of D618- and D657-, no D393-CD20 expression was measured, contrasting with DBCL. These results suggested a CD20 splicing modulation caused by an oncogenesis process rather than viral infection itself. This strengthens the hypothesis of an association between D393-CD20 and oncogenesis. It is known that the SM (Mta, EB2, BMLF1) EBV protein, a viral oncogenic nuclear protein bound to RNA, influences RNA stability, splicing, nuclear export, and translation. This influence facilitates virus replication and persistence in vivo [35]. SM protein is associated with three splice regulators, SF2/ASF (SRSF1), 9G8 (SRSF7), and SRp20 (SRSF3), and antagonizes SRSF3 [36]. Thus, the SM EBV protein may be an actor that regulates CD20 cellular gene expression at the level of alternative splicing.

This work shows a deregulated expression of CD20 variant transcripts in B malignancies that may be useful as a molecular marker to study splicing patterns in order to better classify malignancies, predict resolution of disease, or monitor treatment [12]. In this way, we took advantage of the availability of *sf3b1* mutational status of the BOMP relapsed CLL cohort to evaluate if there is a correlation with CD20 splicing. CLL disease is an interesting model because mutations of *sf3b1*, which encode a critical component of the splicing machinery, are associated with progression and fludarabin-refractoriness [37]. Interestingly, we noticed that patient group with more *cd20* alternative splicing correspond to those with higher *sf3b1* mutation frequency (data not shown). These results should be confirmed with other CLL cohorts, and a potential correlation with other gold standard biomarkers of CLL should be investigated. Moreover, a significant difference of total CD20 splicing between the 2 CLL cohorts at diagnosis (routine CLL cohort *vs* elderly CLL2007-SA, respectively 50 and 11 % stage A) could make this marker an indicator of the stage of the disease progression, which could be useful for CLL stratification.

Alternative CD20 splicing may have consequences on CD20 protein function that may influence BCR/CD20 cell signaling and finally B cell functions. We previously described that D393-CD20 transcript encoded a truncated CD20 protein [22]. Using CD20 immunoprecipitation with an antibody targeting the extracellular domain followed by western blot with the C-terminal CD20 specific antibody, we have already demonstrated that D393-CD20 protein is associated with wtCD20. According to the predicted sizes of other putative proteins, they could also be associated with wtCD20 since the sizes match additional bands observed on western blot (Additional file 1: Figure S4). Subcellular division of transfected cells with the D393-CD20 coding sequence revealed that the variant protein is found mainly in the membrane fraction, although the main part of the transmembrane coding sequence is missing. This result strongly suggests an association between wtCD20 and D393-CD20 protein. Finally, lipid raft isolation showed the presence of D393-CD20 and wtCD20 already within the lipid rafts. All of these observations suggest a possible involvement of proteins encoded by *cd20* alternative variants in BCR signaling or calcium flux, both putative functions of CD20 protein [38].

Another consequence of the CD20 splicing is the production of in-frame mRNA that could be translated into new proteins and could thus participate in the tumoral edition by generating neo-epitopes that could be targeted in anti-tumoral vaccine strategies [39, 40]. Concerning CD20 alternative splice variants, we have demonstrated that the 20mer D393-CD20 peptide spanning the splicing site might be targeted by the immune system, and we have shown that D393-CD20—specific CD4 Th1 clones could directly recognize malignant B cell lines and kill autologous lymphoma B cells, indicating that D393-CD20—derived epitopes are naturally processed and presented on tumor cells [41]. Additional CD20 alternative variants may also be new tumoral antigens that could be targeted by a redirected immune system, such as transgenic T cell receptors.

These observations may be useful for the development of new immunotherapies applied to patients refractory to conventional (chemotherapy) or targeted treatments (anti-CD20, Ibrutinib, iBTK).

In conclusion, the discovery of new alternative CD20 transcript variants makes them of interest as molecular indicators to investigate in further studies, particularly given the involvement of some of them in EBV transformation, their association with oncogenesis rather than non-oncogenic B diseases, their differential expression in B malignancies, and correlation with CLL stage and some predictive CLL markers. Overall, these findings need to be confirmed by larger prospective trials in order to fully validate CD20 transcript variant as molecular markers of oncogenesis.

## Methods

### Patients, biological samples, and cell lines

Master cell banks of human and mouse cell lines were prepared from cells from the DSMZ or ATCC cell banks. Working cell cultures were then established, and cells were cultured in RPMI 1640 or DMEM with 10 % fetal calf serum. STR profiling identification was performed regularly.

Peripheral blood samples were selected from cases of hematologic B cell disease: B-CLL, follicular lymphoma (FL), mantle cell lymphoma (MCL), diffuse large B cell lymphoma (DLBCL) and cutaneous B cell lymphoma (CBCL), multiple myeloma (MM), marginal zone lymphoma, non-Hodgkin lymphoma (NHL), or autoimmune disease (rheumatoid arthritis), as well as infectious mononucleosis (IMN). In addition, EBV-reactivated samples collected from renal, lung, or hematopoietic allografts were screened. Samples were collected from diagnostic assessment or clinical trials or from a blood bank for the healthy PBMCs.

EBV-derived B lymphoblastoid cell lines (BLCLs) were established from healthy donor PBMCs. PBMCs were transformed with EBV supernatant in X-VIVO medium with cyclosporine A at 1 µg/ml for 2 days and maintained in culture for at least 10 days, until an immortalized B cell line was obtained.

CLL samples were collected from three different cohorts of patients: PBMCs collected at diagnosis for routine analysis (CHU Toulouse, France); CD19+ immunomagnetic-purified B cells (whole human blood CD19 MicroBeads, Miltenyi Biotec) from CLL patient samples, stage B and C, included within the CLL2007-SA (for elderly patients older than 65 years); and patients included in the ICLL01 BOMP clinical trial (relapsed or refractory CLL stages A, B, or C with active disease or after 1–3 previous lines including at least one line with fludarabine), both initiated by the GOELAMS/GCFLLC-MW intergroup. Written informed consent was obtained according to institutional protocol and approbation of the Ethic Committee (Comité de protection des personnes: CPP-Est, France).

### Western blotting

Cells were lysed in sample buffer (2 % sodium dodecyl sulfate (SDS) in 125 mM Tris HCl, pH 6.8). An equivalent protein amount, extracted from 1 × 10^7^ to 8 × 10^7^ cells, was separated by electrophoresis on 12 % SDS–polyacrylamide gels and transferred to Polyvinylidene difluoride (PDVF) membranes (GE Healthcare).

Blots were then blocked for 1 h in 6 % milk before incubation with specific antibodies as follows: rabbit anti-human CD20 specific to the COOH-terminal region [22] (Thermo Scientific) and rabbit anti-actin (#8457L, Cell Signaling). Blotted proteins were detected and quantified on a bioluminescence imager and BIO-1D advanced software (Vilber-Lourmat) after blots were incubated with a horseradish peroxidase–conjugated appropriate secondary antibody (Beckman Coulter).

### Molecular studies: RNA isolation, reverse transcription, cloning, real-time quantification, and Sanger cycle sequencing

Total RNA was extracted using the RNeasy Total RNA Isolation kit (Qiagen, Courtaboeuf, France), following manufacturer protocols. One microgram of total RNA was used as template for cDNA synthesis performed using a high-capacity RNA to cDNA kit (Applied Biosystem, Courtaboeuf, France).

Genomic DNA was extracted using a DNeasy blood or tissue kit (Qiagen, Courtaboeuf, France) or the salting out method. Briefly, cells were lysed by TES buffer supplemented by SDS 20 % and proteinase K 0.5 mg/ml. Proteins were then precipitated in a saturated NaCl solution and centrifuged, and DNA then was precipitated using ethanol.

Qualitative RT-PCR was performed using the MyTaq DNA polymerase ready-to-use master mix (Bioline, France) and specific primers. PCR products were analyzed by agarose gel electrophoresis followed by ultraviolet detection. When useful, PCR products were gel purified, cloned within pCR^®^ 2.1-TOPO^®^ TA vector (Life Technologies), and Sanger forward and reverse sequenced using M13 primers. Purified sequencing products were run on an ABI-3130 DNA analyzer and analyzed using sequencing analysis v5.2 software (Applied Biosystems). Sequences were aligned against the wild-type (wt)CD20 coding sequence using the Bioedit v7.1 software.

Quantitative RT-PCR (RT-qPCR) was performed using splice variant–specific primers and bi-fluorescence probes. cDNA was amplified with TaqMan Universal Master Mix with UNG (Applied Biosystem, Courtaboeuf, France) using a standard two-step amplification program (10 s at 95° and 1 min at 60°).

CD20 variant transcript copy number was assessed by RT-qPCR against a plasmid dilution curve. All PCR samples were normalized to ABL copy number. The proportion of each CD20 transcript variant was calculated against all CD20 isoforms.

PCR conditions, sizes of PCR products, and names and sequences of primers are described in Additional file 1: Table S1. Schematic localizations of all PCR primers and bi-fluorescent probes are provided in Additional file 1: Figure S1.

### Cell transfection and packaging cell line production

The wtCD20 coding sequence was first cloned into a pcDNA3.1-GFP (green fluorescent protein) mammalian expression vector. To restore canonical splice sites within the cDNA coding sequence, intron 5 (int5) was previously reintroduced into pcDNA3.1-GFP-wtCD20 to generate pcDNA3.1-GFP-int5-CD20, theoretically producing D618-CD20 transcripts. Intron 6 (int6) was then inserted into pcDNA3.1-GFP-int5-CD20 to generate pcDNA3.1-GFP-int5– and 6-CD20–expressing D657-CD20 transcripts. Finally, reintroduction of introns 3 (int3) and 6 within the wtCD20 sequence allowed expression of D480-CD20 mRNA from pcDNA3.1-GFP-int3 and the 6-CD20 vector. All of these vectors were amplified into JM105 bacteria. The HT1080 cell line was then transfected by these vectors using the Lipofectamine transfection kit (Life Technologies) to produce transiently expressing cell lines.

In addition to the D393-CD20 packaging cell line, previously produced, wtCD20, D657-CD20, and D618-CD20 coding sequences were inserted into the retroviral pBABE-GFP vector (Addgene, UK). The PG13 packaging cell line was transfected by the pBABE-GFP-wtCD20, pBABE-GFP-D657-CD20, and pBABE-GFP-D618-CD20 vectors using the Lipofectamine transfection kit (Life Technologies). Supernatants were then collected at 48, 72, and 96 h to produce HT1080-transduced cells, cultured and selected in puromycin-containing medium. The percentage of stably transduced cells was controlled by assessing GFP expression by flow cytometry.

### Splice site prediction and statistical computational analysis

Splicing sites were identified using online splice site prediction tools such as SpliceProt prediction [24] (http://www.spliceport.org.) or ASSP Prediction [25] (http://wangcomputing.com/assp/). Statistical analysis was performed using the Χ^2^ test.

## Additional file

**Additional file 1: Table S1.** Table of primers used for wtCD20 and transcript variant detection (RT‐PCR) as well as realtime PCR quantification (RT‐qPCR). Specific annealing temperature and PCR product size in bp are given for RT‐PCR. ABL PCR was used for control gene expression quantification. **Table S2:** Characteristics (n, genders, Binet score, biological parameters, mutational status) of the three CLL patient cohorts. NA: not available. **Figure S1:** Schematic representation of wtCD20 and transcript variants. Qualitative PCR primers as well as quantitative primers forward (→) and reverse (←) and bi‐fluorescent probes (•–•) are localized up and down, respectively, on the different transcripts. **Figure S2:** wtCD20 coding sequence (NCBI‐GenBank NM152866.2) given as reference as well as D393‐CD20, previously described [22] shown in blue. The 4 new identified coding sequences of the CD20 alternative transcripts are also in blue. **Figure S3:** Alignment of the newly discovered sequences against the wtCD20 coding sequence using the BioEdit v7.1 software, which allowed precise identification of junction sequence regions. **Figure S4: a/**CD20 immunoprecipitation (IP) was performed using an antibody specific to an extracellular epitope of human CD20 (#302302, Biolegend) and western blot detection with the cterminal human CD20 Rabbit Polyclonal antibody (#E2562, Thermofischer) **b/**Subcellular fractions [Membrane (M), Cytoplasm (C), Nucleus (N)] obtained from 293 cells transfected with a lentiviral vector pFIV‐D393‐CD20 or pFIV‐wtCD20 were subjected to western blot analysis using c‐terminal CD20 or actin (for protein loading control) antibodies. Blotted proteins were detected and quantified on a bioluminescence imager with BIO‐1D advanced software (Wilber‐Lourmat) after incubation of blots with a horseradish peroxidase–conjugated appropriate secondary antibody (Beckman Coulter). **c/**Lipid raft isolation by ultra‐centrifugation on sucrose density gradient. Fractions 1 to 4 (10 % to 40 % of sucrose density) respectively harvested after centrifugation were subjected to c‐terminal anti‐CD20 western blotting. Actin and Flotillin‐2 antibody staining was used as protein‐loading and lipid‐raft control, respectively.
