# Erratum to: New CD20 alternative splice variants: molecular identification and differential expression within hematological B cell malignancies

**DOI:** 10.1186/s40164-016-0038-1

**Published:** 2016-04-14

**Authors:** Clémentine Gamonet, Elodie Bole-Richard, Aurélia Delherme, François Aubin, Eric Toussirot, Francine Garnache-Ottou, Yann Godet, Loïc Ysebaert, Olivier Tournilhac, Caroline Dartigeas, Fabrice Larosa, Eric Deconinck, Philippe Saas, Christophe Borg, Marina Deschamps, Christophe Ferrand

**Affiliations:** 1 INSERM UMR1098, Établissement Français du Sang Bourgogne Franche Comté, Université de Franche-Comté, SFR FED4234, 25020 Besançon, France; 2 EA3181 et Service de Dermatologie, Université de Franche Comté, CHU de Besançon, Besançon, France; 3 CHRU, Department of Rheumatology, Université de Franche-Comté EA 4266, INSERM CIC-1431, 25000 Besançon, France; 4 EA 4266, Université de Franche-Comté, Besançon, France; 5 Inserm U1037, Université Toulouse 3-ERL CNRS, CHU Purpan, Toulouse, France; 6 Hématologie Clinique, CHU Estaing, 1 Place Lucie Aubrac, 63003 Clermont-Ferrand Cedex 1, France; 7 Hematologie, CHU Bretonneau, Tours, France; 8 Hematology Department, CHU Jean Minjoz, 25020 Besançon, France; 9 Laboratoire de Thérapeutique Immuno-Moléculaire et cellulaire des cancers, INSERM UMR1098, Etablissement Français du Sang–Bourgogne/Franche-Comté, 8, rue du Docteur Jean-François-Xavier Girod, 25020 Besançon Cedex, France

## Erratum to: Exp Hematol Oncol (2016) 5:7 DOI 10.1186/s40164-016-0036-3

Unfortunately, the original version of this article [1] contained an error. The author Caroline Dartigeas’ name was inverted. The correct presentation is as noted here, Caroline Dartigeas. There will be an update to amend this as well as this erratum.
