# Supplementary material for: New CD20 alternative splice variants: molecular identification and differential expression within hematological B cell malignancies
Source: Exp Hematol Oncol. 2016 Mar 1;5:7. doi: 10.1186/s40164-016-0036-3 (PMC4774009; doi:10.1186/s40164-016-0036-3)

Supplementary Data

New CD20 alternative splice variants: molecular identification and differential expression within hematological B-cell malignancies.

Clémentine Gamonet1, Elodie Bole-Richard 1, Aurélia Delherme1, François Aubin2, Eric Toussirot2,3, Francine Garnache-Ottou12,, Yann Godet1,2, Loïc Ysbaert4, Olivier Tournilhac5, Dartigeas Caroline6 Fabrice Larosa1,7, Eric Deconinck1,2.7, Philippe Saas1,2, Christophe Borg1,2, Marina Deschamps1 & Christophe Ferrand1

Supplementary data

Table S1

- **Qualitative PCR (RTPCR)**

PCR name	Primer Forward		Primer Reverse		Annealing Temperature	Size of PCR product(s) (bp)
	Name	Sequence (5'-3')	Name	Sequence (5'-3')		
fl-CD20	START	ATGACAACACCCAGAAATTC	STOP	TTAAGGAGAGCTGTCATTTTCT	58°C	894, 657, 618, 480, 393, 177
D657	Fw-D657-spe	TCCAGGAAGTGTTT**G/G**GCAT	STOP	TTAAGGAGAGCTGTCATTTTCT	58°C	337
D618	Fw-C2	GGGATCTATGCACCCATCTGTGTGA	Rv-D618spe	CCAGCTATTACAAGTT**C/C**AAAC	60°C	130
D480	Fw-C1	CGGCAGAGCCAATGAAAGG	Rv-D480spe	CACTGACAAAATGC**C/C**CCCA	58°C	137
D393	Fw-D393-spe	GATGTCTTCACT**G/G**AACT	STOP	TTAAGGAGAGCTGTCATTTTCT	58°C	295
D177	START	**ATG**ACAACACCCAGAAATTC	Rv-D177-spe	ATTGGTGAGGATT**C/C**CTCA	55°C	155

Underline characters represent start and stop codons. (/) denotes splice junction. Fw: Forward primers, Rv: Reverse primers. Spe : splice variant specific primers

- **Real time quantitative PCR (RTqCPR)**

qPCR name	Primer Forward		Primer Reverse		Probe		Te
	Name	Sequence (5'-3')	Name	Sequence (5'-3')	Name	Sequence (5'FAM -3'TAMRA)	
wtCD20	TQM-T1-CD20-Fw	ATTATTTCCGGATCACTCCT	TQM-CD20wt	TAATGAATTCATTGAGCCTC	T1-CD20	CAACGGAGAAAAACTCCAGG	
D657	TQM-T1-CD20-Fw	ATTATTTCCGGATCACTCCT	***TQM-Rv-D657-Spe***	TTTGGGCATTTTGTCAGTGA	T1-CD20	CAACGGAGAAAAACTCCAGG	
D618	TQM-T1-CD20-Fw	ATTATTTCCGGATCACTCCT	***TQM-Rv-D618-Spe***	TTTGGAACTTGTAATAGCTG	T1-CD20	CAACGGAGAAAAACTCCAGG	
D480	TQM-T2-CD20-Fw	CCTATTGCTATGCAATCTGG	***TQM-Rv-D480-Spe***	GGGGGGCATTTTGTCAGTGA	T2-CD20	AACCACTCTTCAGGAGGATG	
D393	TQM-T2-CD20-Fw	CCTATTGCTATGCAATCTGG	***TQM-Rv-D393-Spe***	ACTGGAACTTGTAATAGCTG	T2-CD20	AACCACTCTTCAGGAGGATG	
D177	START	ATGACAACACCCAGAAATTC	STOP	TTAAGGAGAGCTGTCATTTTCT	***D177-spe***	AAAGCTTCTTCATGAGGGAATC	
Control (Abl)	*Abl-Fw*	*TGGAGATAACACTCTAAGCATAACTAAAGGT*	*Abl-Rv*	*GATGTAGTTGCTTGGGACCCA*	*TQM-Abl*	*CCATTTTTGGTTTGGGCTTCACACCATT*	

Underline characters represent start and stop codons. Fw: Forward primers, Rv: Reverse primers. Spe (in bold): splice variant specific primers or bi-fluorescent probe.

Table S2 : Characteristic of the 3 CLL patients cohorts

		CLL2007-SA	CLL01 BOMP	CLL routine
**n**		54	70	59
**Media age (years)**		70.7	64	64
**Gender (M/F)**		0.6/0.4	0.79/0.21	0.60/0.40
Binet Score	A : n, (%)	19/51 (37.2)	6/53 (11)	30/59 (50.8)
	B : n, (%)	32/51 (62.7)	27/53 (51)	17/59 (28.8)
	C : n, (%)	0/51 (0)	20/53 (37.7)	12/59 (20.3)
Mutationnal status	IgVH (n/total) %	(24/44) 54.5%	(45/50) 90%	(24/58) 41%
	SF3B1 (n/total) %	NA	(13/53) 24%	(2/60) 3%
	Notch (n/total) %	NA	(7/53) 13%	(8/60) 13%
	TP53 (n/total) %	NA	(17/52) 32.7%	(5/17) 11%
[illegible] al Parame[illegible]	Beta2-microglobulin	NA	(27/53) 50.9	NA
	CD38 positive (>20%)	NA	(37/53) 69.8	NA

Figure S1 : Schematic design of RTPCR or RTpQCR

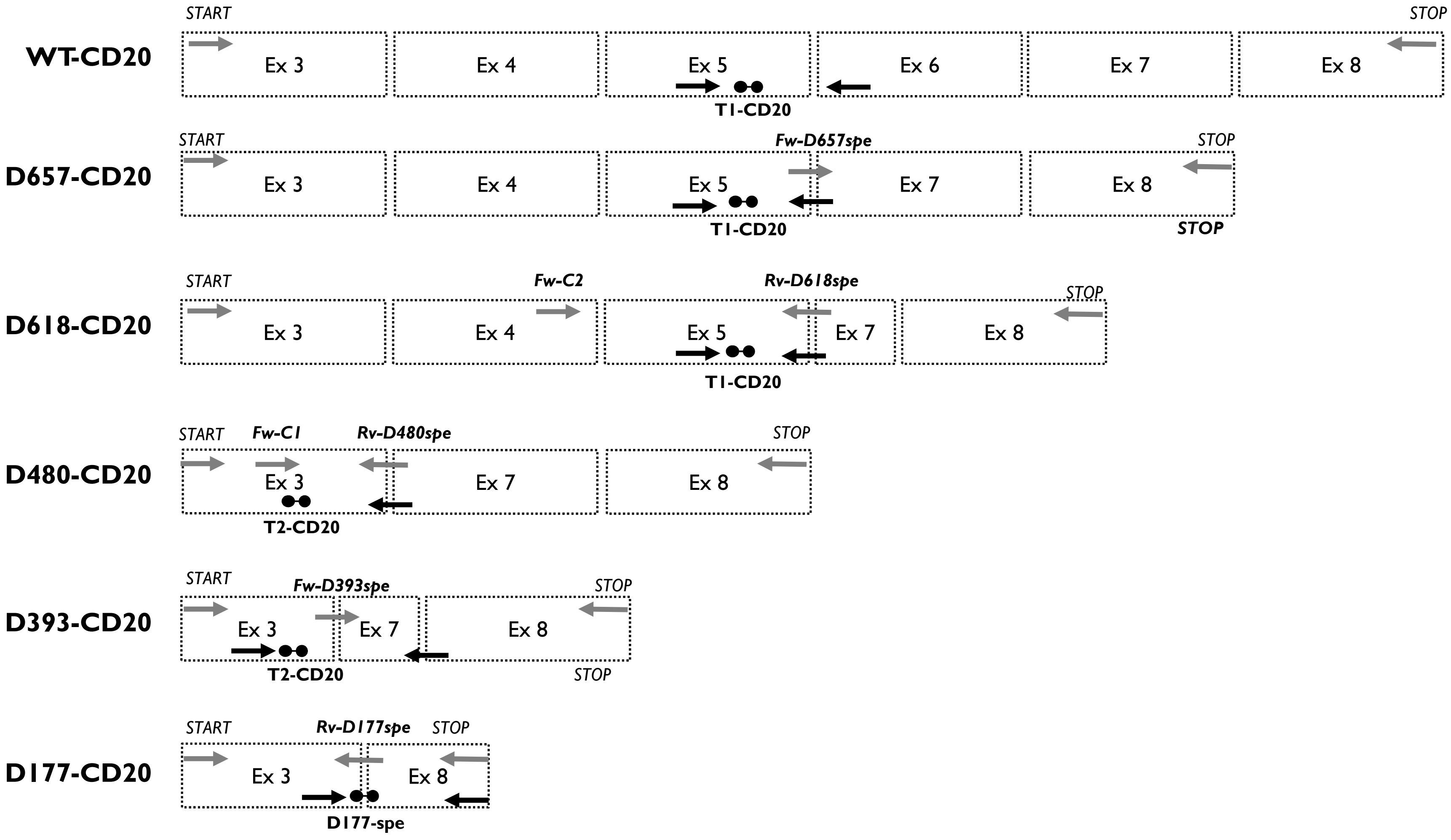

Figure S2 : sequences of wt-CD20 and D393 coding sequences (in blue) and the 4 newly Sanger-sequencing identified sequences (in black)

>wt-CD20 coding Sequence (ref NCBI-Genbank NM152866.2)

ATGACAACACCCAGAAATTCAGTAAATGGGACTTTCCCGGCAGAGCCAATGAAAGGCCCTATTGCTATGCAATCTGGTCCAAAACCACTCTTCAGGAGGATGTCTTCACTG**GT**GGGCCCCACGCAAAGCTTCTTCATGAGGGAATCTA
AGACTTTGGGGGCTGTCCAGATTATGAATGGGCTCTTCCACATTGCCCTGGGGGGTCTTCTGATGATCCCAGCAGGGATCTATGCACCCATCTGTGTGACTGTGTGGTACCCTCTCTGGGGAGGCATTATGTATATTATTTCCGGATC
ACTCCTGGCAGCAACGGAGAAAAACTCCAGGAAGTGTTTG**GT**CAAAGGAAAAATGATAATGAATTCATTGAGCCTCTTTGCTGCCATTTCTGGAATGATTCTTTCAATCATGGACATACTTAATATTAAAATTTCCCATTTTTTAAAA
ATGGAGAGTCTGAATTTTATTAGAGCTCACACACCATATATTAACATATACAACTGTGAACCAGCTAATCCCTCTGAGAAAAACTCCCCATCTACCCAATACTGTTACAGCATACAATCTCTGTTCTTGGGCATTTTGTCAGTGATGC
TGATCTTTGCCTTCTTCCAGGAACTTGTAATAGCTGGCATCGTTGAGAATGAATGGAAAAGAACGTGCTCCAGACCCAAATCTAACATAGTTCTCCTGTCAGCAGAAGAAAAAAAAGAACAGACTATTGAAATAAAAGAAGAAGTGGT
TGGGCTAACTGAAACATCTTCCCAACCAAAGAATGAAGAAGACATTGAAATTATTCCAATCCAAGAAGAGGAAGAAGAAGAAACAGAGACGAACTTTCCAGAACCTCCCCAAGATCAGGAATCCTCACCAATAGAAAATGACAGCTCT
CCT**TAA**

>D393-CD20 sequence (Henry et al, 2010)

ATGACAACACCCAGAAATTCAGTAAATGGGACTTTCCCGGCAGAGCCAATGAAAGGCCCTATTGCTATGCAATCTGGTCCAAAACCACTCTTCAGGAGGATGTCTTCACTGGAACTTGTAATAGCTGGCATCGTTGAGAATGAATGGA
AAAGAACGTGCTCCAGACCCAAATCTAACATAGTTCTCCTGTCAGCAGAAGAAAAAAAAGAACAGACTATTGAAATAAAAGAAGAAGTGGTTGGGCTAACTGAAACATCTTCCCAACCAAAGAATGAAGAAGACATTGAAATTATTCC
AATCCAAGAAGAGGAAGAAGAAGAAACAGAGACGAACTTTCCAGAACCTCCCCAAGATCAGGAATCCTCACCAATAGAAAATGACAGCTCTCCT**TAA**

>D657-CD20 sequence

ATGACAACACCCAGAAATTCAGTAAATGGGACTTTCCCGGCAGAGCCAATGAAAGGCCCTATTGCTATGCAATCTGGTCCAAAACCACTCTTCAGGAGGATGTCTTCACTGGTGGGCCCCACGCAAAGCTTCTTCATGAGGGAATCTA
AGACTTTGGGGGCTGTCCAGATTATGAATGGGCTCTTCCACATTGCCCTGGGGGGTCTTCTGATGATCCCAGCAGGGATCTATGCACCCATCTGTGTGACTGTGTGGTACCCTCTCTGGGGAGGCATTATGTATATTATTTCCGGATC
ACTCCTGGCAGCAACGGAGAAAAACTCCAGGAAGTGTTTGGGCATTTTGTCAGTGATGCTGATCTTTGCCTTCTTCCAGGAACTTGTAATAGCTGGCATCGTTGAGAATGAATGGAAAAGAACGTGCTCCAGACCCAAATCTAACATA
GTTCTCCTGTCAGCAGAAGAAAAAAAAGAACAGACTATTGAAATAAAAGAAGAAGTGGTTGGGCTAACTGAAACATCTTCCCAACCAAAGAATGAAGAAGACATTGAAATTATTCCAATCCAAGAAGAGGAAGAAGAAGAAACAGAGA
CGAACTTTCCAGAACCTCCCCAAGATCAGGAATCCTCACCAATAGAAAATGACAGCTCTCCT**TAA**

>D618-CD20 sequence

ATGACAACACCCAGAAATTCAGTAAATGGGACTTTCCCGGCAGAGCCAATGAAAGGCCCTATTGCTATGCAATCTGGTCCAAAACCACTCTTCAGGAGGATGTCTTCACTGGTGGGCCCCACGCAAAGCTTCTTCATGAGGGAATCTA
AGACTTTGGGGGCTGTCCAGATTATGAATGGGCTCTTCCACATTGCCCTGGGGGGTCTTCTGATGATCCCAGCAGGGATCTATGCACCCATCTGTGTGACTGTGTGGTACCCTCTCTGGGGAGGCATTATGTATATTATTTCCGGATC
ACTCCTGGCAGCAACGGAGAAAAACTCCAGGAAGTGTTTGGAACTTGTAATAGCTGGCATCGTTGAGAATGAATGGAAAAGAACGTGCTCCAGACCCAAATCTAACATAGTTCTCCTGTCAGCAGAAGAAAAAAAAGAACAGACTATT
GAAATAAAAGAAGAAGTGGTTGGGCTAACTGAAACATCTTCCCAACCAAAGAATGAAGAAGACATTGAAATTATTCCAATCCAAGAAGAGGAAGAAGAAGAAACAGAGACGAACTTTCCAGAACCTCCCCAAGATCAGGAATCCTCAC
CAATAGAAAATGACAGCTCTCCT**TAA**

>D480-CD20 sequence

ATGACAACACCCAGAAATTCAGTAAATGGGACTTTCCCGGCAGAGCCAATGAAAGGCCCTATTGCTATGCAATCTGGTCCAAAACCACTCTTCAGGAGGATGTCTTCACTGGTGGGCCCCACGCAAAGCTTCTTCATGAGGGAATCTA
AGACTTTGGGGGGCATTTTGTCAGTGATGCTGATCTTTGCCTTCTTCCAGGAACTTGTAATAGCTGGCATCGTTGAGAATGAATGGAAAAGAACGTGCTCCAGACCCAAATCTAACATAGTTCTCCTGTCAGCAGAAGAAAAAAAAGA
ACAGACTATTGAAATAAAAGAAGAAGTGGTTGGGCTAACTGAAACATCTTCCCAACCAAAGAATGAAGAAGACATTGAAATTATTCCAATCCAAGAAGAGGAAGAAGAAGAAACAGAGACGAACTTTCCAGAACCTCCCCAAGATCAG
GAATCCTCACCAATAGAAAATGACAGCTCTCCT**TAA**

>D177-CD20 sequence

ATGACAACACCCAGAAATTCAGTAAATGGGACTTTCCCGGCAGAGCCAATGAAAGGCCCTATTGCTATGCAATCTGGTCCAAAACCACTCTTCAGGAGGATGTCTTCACTGGTGGGCCCCACGCAAAGCTTCTTCATGAGGGAATCCT
CACCAATAGAAAATGACAGCTCTCCT**TAA**

Figure S3 : alignment of the 4 newly Sanger-sequenced sequences against CD20 reference sequence (NCBI-Genebank NM152866.2)

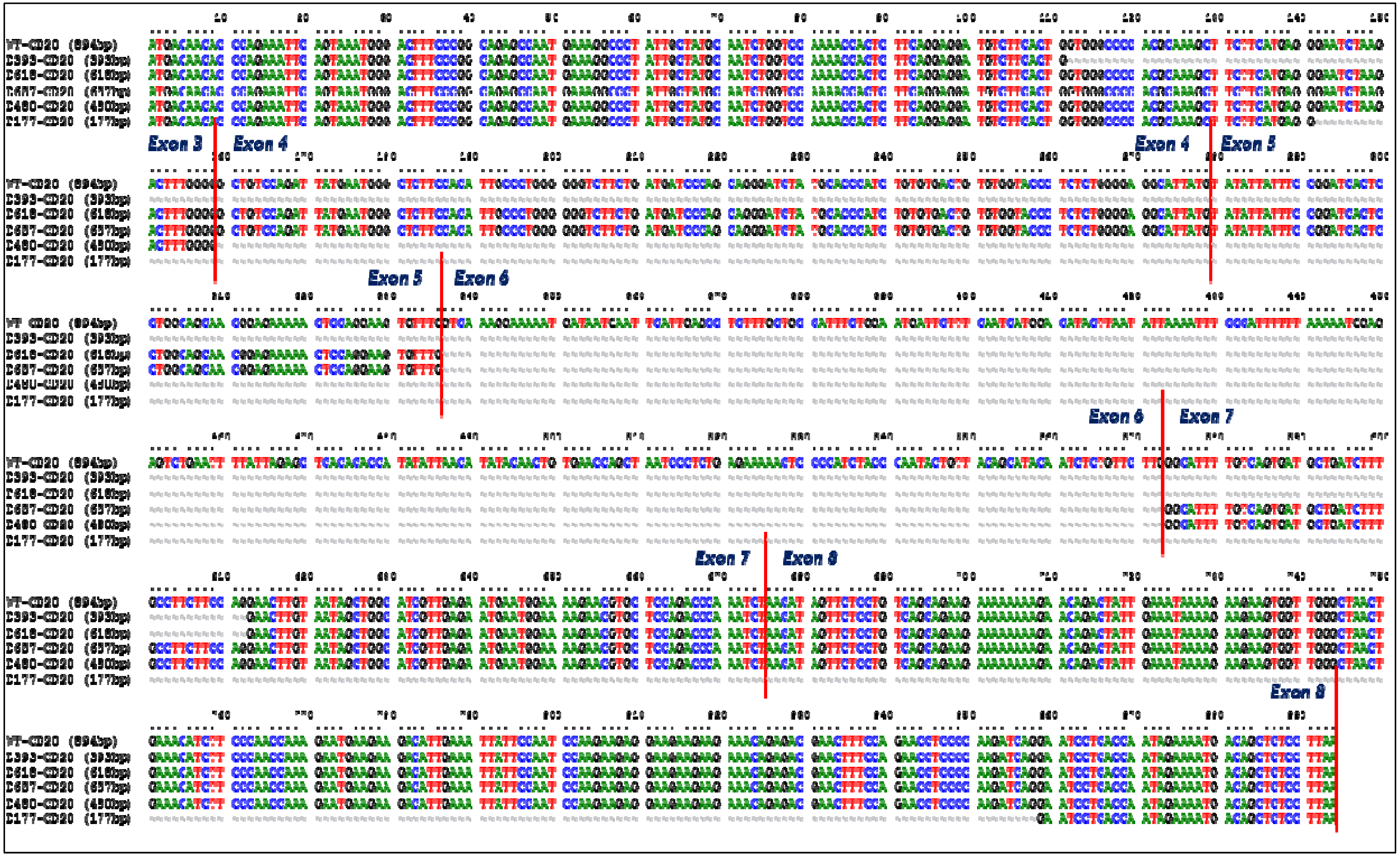

Alignment provided by the BioEdit© Software v7,1

Figure S4

a

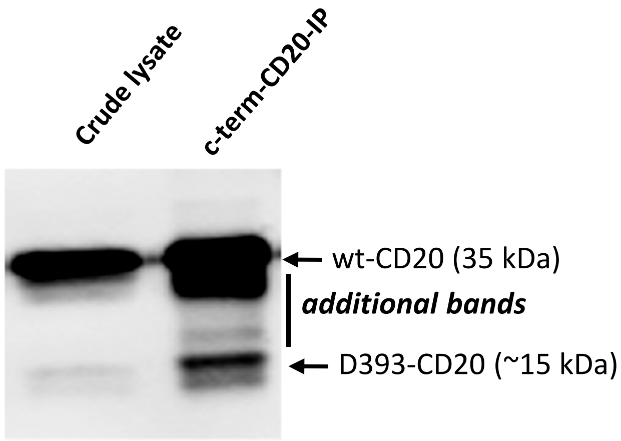

b

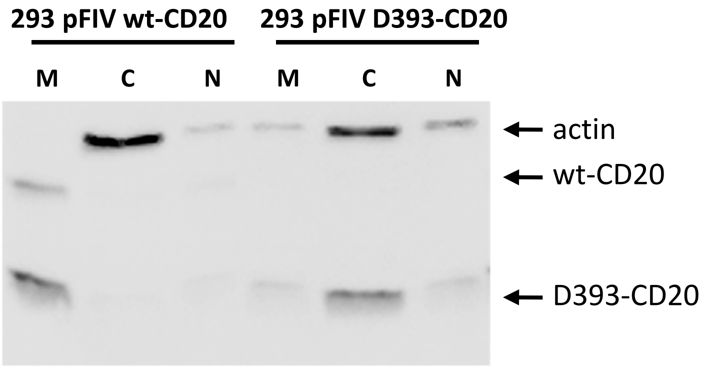

c

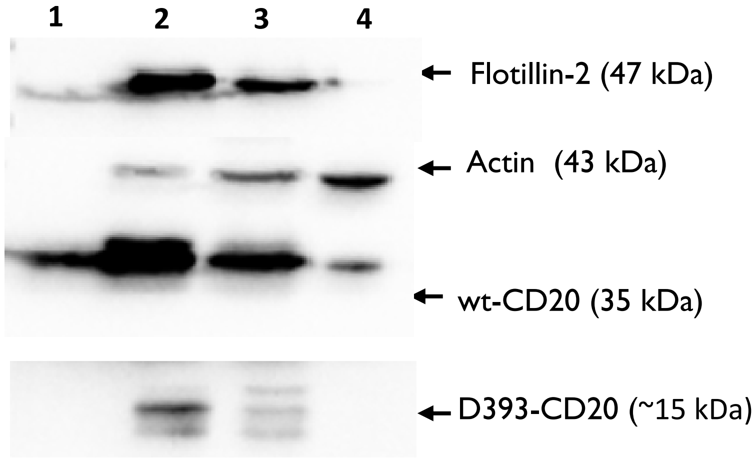

Supplement: Supplementary file 1 — Additional file 1: Table S1. Table of primers used for wtCD20 and transcript variant detection (RT‐PCR) as well as realtime PCR quantification (RT‐qPCR). Specific annealing temperature and PCR product size in bp are given for RT‐PCR. ABL PCR was used for control gene expression quantification. Table S2: Characteristics (n, genders, Binet score, biological parameters, mutational status) of the three CLL patient cohorts. NA: not available. Figure S1: Schematic representation of wtCD20 and transcript variants. Qualitative PCR primers as well as quantitative primers forward (→) and reverse (←) and bi‐fluorescent probes (•–•) are localized up and down, respectively, on the different transcripts. Figure S2: wtCD20 coding sequence (NCBI‐GenBank NM152866.2) given as reference as well as D393‐CD20, previously described [22] shown in blue. The 4 new identified coding sequences of the CD20 alternative transcripts are also in blue. Figure S3: Alignment of the newly discovered sequences against the wtCD20 coding sequence using the BioEdit v7.1 software, which allowed precise identification of junction sequence regions. Figure S4: a/CD20 immunoprecipitation (IP) was performed using an antibody specific to an extracellular epitope of human CD20 (#302302, Biolegend) and western blot detection with the cterminal human CD20 Rabbit Polyclonal antibody (#E2562, Thermofischer) b/Subcellular fractions [Membrane (M), Cytoplasm (C), Nucleus (N)] obtained from 293 cells transfected with a lentiviral vector pFIV‐D393‐CD20 or pFIV‐wtCD20 were subjected to western blot analysis using c‐terminal CD20 or actin (for protein loading control) antibodies. Blotted proteins were detected and quantified on a bioluminescence imager with BIO‐1D advanced software (Wilber‐Lourmat) after incubation of blots with a horseradish peroxidase–conjugated appropriate secondary antibody (Beckman Coulter). c/Lipid raft isolation by ultra‐centrifugation on sucrose density gradient. Fractions 1 to 4 (10 % to 40 % of su [file 40164_2016_36_MOESM1_ESM.pdf]
